# Cardiometabolic profiles and proteomics associated with obesity phenotypes in a longitudinal cohort of young adults

**DOI:** 10.1038/s41598-024-57751-2

**Published:** 2024-03-28

**Authors:** Jiawen Liao, Jesse A. Goodrich, Wu Chen, Chenyu Qiu, Jiawen Carmen Chen, Elizabeth Costello, Tanya L. Alderete, Lida Chatzi, Frank Gilliland, Zhanghua Chen

**Affiliations:** 1 Department of Public and Population Health Sciences, Keck School of Medicine of the University of Southern California, Los Angeles, CA 90032 USA; 2 Department of Integrative Physiology, University of Colorado, Boulder, CO USA

**Keywords:** Epidemiology, Biomarkers, Molecular medicine, Dyslipidaemias, Metabolic syndrome, Obesity

## Abstract

To assess cardiometabolic profiles and proteomics to identify biomarkers associated with the metabolically healthy and unhealthy obesity. Young adults (N = 156) enrolled were classified as not having obesity, metabolically healthy obesity (MHO) and metabolically unhealthy obesity (MUHO) based on NCEP ATP-III criteria. Plasma proteomics at study entry were measured using Olink Cardiometabolic Explore panel. Linear regression was used to assess associations between proteomics and obesity groups as well as cardiometabolic traits of glucose, insulin, and lipid profiles at baseline and follow-up visits. Enriched biological pathways were further identified based on the significant proteomic features. Among the baseline 95 (61%) and 61 (39%) participants classified as not having obesity and having obesity (8 MHO and 53 MUHO), respectively. Eighty of the participants were followed-up with an average 4.6 years. Forty-one proteins were associated with obesity (FDR < 0.05), 29 of which had strong associations with insulin-related traits and lipid profiles (FDR < 0.05). Inflammation, immunomodulation, extracellular matrix remodeling and endoplasmic reticulum lumen functions were enriched by 40 proteins. In this study population, obesity and MHO were associated with insulin resistance and dysregulated lipid profiles. The underlying mechanism included elevated inflammation and deteriorated extracellular matrix remodeling function.

## Introduction

Obesity epidemic is a serious health problem affecting more than 40% of population in the United States^1^, and is associated with the development of various diseases such as cardiovascular disease, type-2 diabetes and cancer^2–5^. However, it has been documented that a group of the population with obesity does not develop cardiometabolic disorder, referred to as metabolically healthy obesity (MHO) compared to the metabolically unhealthy obesity (MUHO) population who progress to adverse cardiometabolic disorders^2,6^. It is estimated that MHO has a prevalence of 20–40% based on different criteria in the United States^6^ and its overall prevalence is largely influenced by age, race/ethnicity, and physical activity levels^2,7^. While some evidence pointed out that the MHO phenotype was not associated with an increased risk of mortality^8,9^, other studies indicated that the MHO phenotype could transform to MUHO and bear a higher cardiovascular disease or mortality risk compared to those without obesity^7,10,11^. This indicates that MHO represents an intermediate phenotype within the spectrum of obesity and broader cardiometabolic disorder.

Previous studies suggest that the obesity phenotype provides an opportunity to investigate cardiometabolic traits that distinguish cardiometabolic abnormalities and gain insights of biomarkers and molecular mechanisms. However, the molecular mechanisms and proteomic patterns of obesity phenotypes are not fully understood. To our knowledge, only a few studies investigated proteomic patterns of obesity and MHO based on cross-sectional design and have not assessed the associations with broader cardiometabolic traits^12–14^. In this study, we used a proteomic approach to evaluate differentially expressed cardiometabolic-related proteins among a cohort of 156 young adults. We aimed to understand the biological mechanisms by investigating whether cardiometabolic traits and plasma proteomics differed among obesity phenotypes of non-obesity healthy (NOH), MHO and MUHO.

## Results

### Study cohort characteristics by groups

The characteristics of the study cohort at baseline obesity group (NOH, MHO and MUHO) are listed in the Table 1. In the baseline, the mean (standard deviation, SD) of age was 19.4 (1.3) years, and slightly more than half of study participants (N = 84, 53%) were male. Most participants were non-Hispanic white (N = 100, 64%) or Hispanic white (N = 41, 26%). Most of the participants had parents with education at college or higher levels (N = 97, 62%) and most participants were students (N = 45, 29%) or students with part time jobs (N = 66, 42%). Additionally, most of the participants reported they exercised at least once a week (N = 117, 75%) and did not smoke (N = 153, 86%). The mean (SD) of the DASH nutrition index for participants was 2.28 (1.54). At baseline, there were 156 participants with complete outcome assessment, and were classified as non-obese healthy (NOH, N = 95, 61%), metabolically healthy obesity (MHO, N = 8, 5%) and metabolically unhealthy obesity (MUHO, N = 53, 34%). The obesity traits (BMI and body fat percentage) and abdominal adipose measures showed significant differences between NOH and obesity group (MHO and MUHO), while no significant differences were found between MHO and MUHO (Table 1). Among all participants, 80 participants were followed-up in 4.6 years; and 43 (54%) were NOH, 7 (8%) were MHO and 30 (38%) were MUHO in the baseline, 40 (50%) were NOH, 13 (16%) were MHO and 27 (34%) were MUHO at follow-up (SI Fig. S1).

**Table 1:** Characteristics of study population by obesity group at baseline.

Characteristic	NOH, N = 95	MHO, N = 8	p-value (between NOH and MHO)^1^	MUHO, N = 53	p-value (between MHO and MUHO)^1^	All, N = 156
Sex (N, %)			1		0.47	
Male	53 (56%)	5 (62%)		25 (47%)		83 (53%)
Female	42 (44%)	3 (38%)		28 (53%)		73 (47%)
Age (Mean, SD)	19.4 (1.3)	19.2 (1.0)	0.72	19.3 (1.4)	0.94	19.4 (1.3)
Ethnicity (N, %)			0.35		0.82	
Hispanic white	32 (33%)	1 (12%)		8 (15%)		41 (26%)
Non-Hispanic white	54 (57%)	7 (88%)		39 (74%)		100 (64%)
Others	9 (10%)	0 (0%)		6 (11%)		15 (10%)
Parental education (N, %)			0.14		0.36	
Completed high school	11 (12%)	3 (38%)		8 (15%)		22 (14%)
Less than high school	19 (20%)	2 (25%)		10 (19%)		31 (20%)
Some college or higher	64 (67%)	3 (38%)		30 (57%)		97 (62%)
Unknown	1 (1%)	0 (0%)		5 (9%)		6 (4%)
Working status (N, %)			0.59		0.48	
Fulltime	23 (24%)	2 (25%)		11 (21%)		36 (23%)
Student and part time working	41 (43%)	4 (50%)		21 (40%)		66 (42%)
Student	26 (27%)	1 (13%)		18 (34%)		45 (29%)
Unemployed	5 (5%)	1 (13%)		3 (6%)		9 (6%)
Exercise once a week (N, %)			1		0.25	
Exercise	76 (80%)	7 (88%)		34 (64%)		117 (75%)
No exercise	19 (20%)	1 (12%)		19 (36%)		39 (25%)
Smoke (N, %)			1		1	
No	91 (96%)	8 (100%)		50 (94%)		149 (96%)
Yes	4 (4%)	0 (0%)		3 (6%)		7 (4%)
Nutritional index (Mean, SD)						
DASH	2.34 (1.50)	2.38 (2.15)	0.66	2.18 (1.56)	0.89	2.28 (1.54)
Obesity measures (mean, SD)						
BMI (kg/m^2^)	26.70 (2.12)	34.80 (4.29)	< 0.001	35.12 (4.32)	0.81	29.98 (5.16)
Total body fat (%)	29.05 (8.75)	37.63 (8.13)	0.011	39.41 (6.49)	0.63	33.01 (9.39)
Abdominal MRI measures						
SAAT (L)	3.71 (1.71)	6.52 (2.78)	0.003	7.89 (2.50)	0.16	5.30 (2.86)
VAT (L)	0.89 (0.60)	1.96 (0.89)	< 0.001	2.16 (1.25)	0.90	1.39 (1.07)
HFF (%)	3.04 (1.43)	4.83 (2.72)	0.01	7.07 (4.73)	0.13	4.51 (3.56)

^1^Fisher’s Exact Test and Wilcoxon rank sum test were used.

### Cardiometabolic traits by obesity group

**Figure 1:**
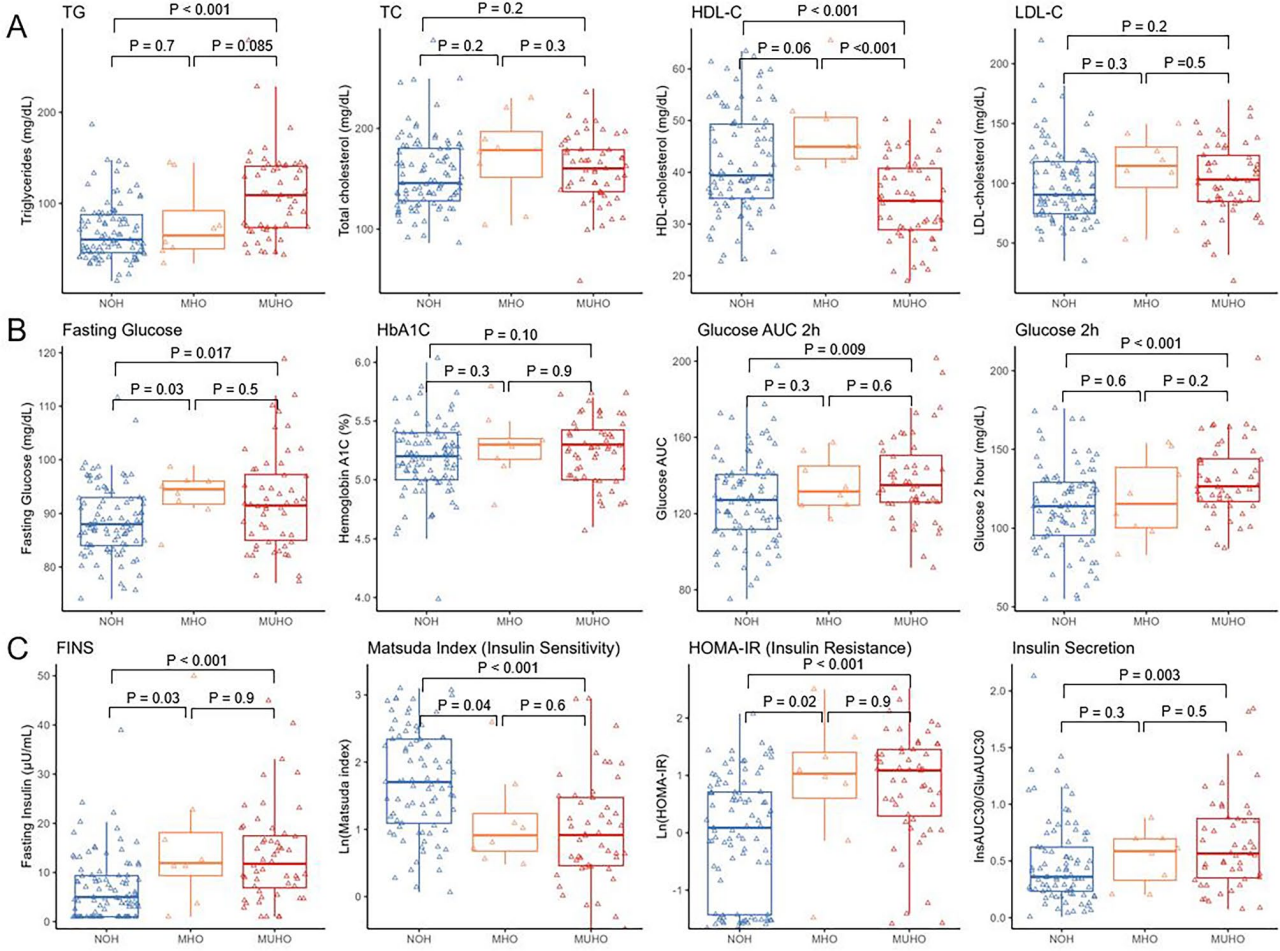
Characteristics of cardiometabolic traits by non-obesity healthy (NOH), metabolically healthy obesity (MHO) and metabolically unhealthy obesity (MUHO) groups at baseline. (**A**) lipid profiles including triglycerides, total cholesterol, HDL-cholesterol and LDL-cholesterol. (**B**) glucose metabolism related outcomes including fasting glucose levels, HbA1C, 2-h glucose under the curve (AUC) in OGTT and 2-h glucose levels in OGTT. (**C**) insulin related outcomes including fasting insulin, insulin sensitivity (Matsuda index), insulin resistance (HOMA-IR) and insulin secretion (InsAUC30/GluAUC30) in OGTT.

Table 2 listed the cardiometabolic traits by obesity group (NOH, MHO and MUHO) in the baseline visit (Fig. 1) and follow-up visit (SI Fig. S2). As expected, participants in the baseline with obesity had deteriorated lipid profiles including higher triglycerides (TG), total cholesterol (TC), higher low density lipoprotein—cholesterol (LDL-C) and lower high density lipoprotein—cholesterol (HDL-C), compared to NOH participants. Additionally, participants in the MUHO group appear to have worsening HDL-C compared to participants in the MHO group. TG showed a strong linear trend for worsening outcomes for MHO and MUHO comparing NOH group, while TC, LDL-C and HDL-C showed some non-monotonic trends (Table 2 and Fig. 1A).

**Table 2:** Characteristics of Lipid Profiles, Glucose-related and Insulin-related of Cardiometabolic Traits at Baseline and Follow-up Visits.

Cardiometabolic outcomes	Baseline, N = 156				Follow up, N = 80			
	NOH, N = 95	MHO, N = 8	MUHO, N = 53	P-value for linear trend	NOH, N = 40	MHO, N = 13	MUHO, N = 27	P-value for linear trend
Triglycerides (mg/dL)^a^	4.13 (0.46)	4.24 (0.51)	4.61 (0.45)^#^	< 0.001	4.16 (0.36)	4.33 (0.28)*	4.76 (0.39)^#†^	< 0.001
Total cholesterol (mg/dL)	154.55 (36.56)	172.11 (45.41)	159.69 (35.32)	0.372	168.00 (36.45)	178.64 (19.91)	189.68 (40.32)^#^	0.014
HDL-C (mg/dL)	41.83 (9.95)	47.90 (8.08)	34.49 (7.97)^#†^	< 0.001	55.48 (9.53)	57.71 (10.99)	47.61 (11.04)^#†^	0.004
LDL-C (mg/dL)	98.93 (31.71)	108.62 (35.18)	103.03 (31.44)	0.425	98.79 (31.11)	105.14 (22.06)	117.14 (34.28)^†^	0.017
Fasting glucose (mg/dL)	88.51 (6.61)	93.38 (4.53)*	95.08 (22.64)^#^	0.007	90.93 (5.60)	90.61 (6.40)	98.81 (8.02)^#†^	< 0.001
HbA1c (%)	5.19 (0.30)	5.29 (0.29)	5.36 (0.81)	0.053	5.14 (0.26)	5.16 (0.36)	5.37 (0.28)^#†^	0.002
Glucose AUC 2-h	127.51 (23.10)	135.19 (14.36)	142.62 (38.64)^#^	0.004	126.90 (15.21)	133.92 (25.97)	147.00 (25.29)^#^	< 0.001
Glucose 2-h (mg/dL)	113.21 (26.81)	119.12 (25.94)	137.24 (47.89)^#^	< 0.001	108.63 (27.24)	120.85 (32.34)	142.19 (39.47)^#†^	< 0.001
Fasting Insulin (FINS) (µU/mL)	6.89 (6.52)	16.13 (15.27)*	16.04 (20.81)^#^	< 0.001	7.40 (6.14)	11.92 (8.84)*	22.75 (12.09)^#†^	< 0.001
Matsuda index ^a^	7.32 (5.46)	4.01 (3.95)*	3.75 (3.95)^#^	< 0.001	5.74 (2.72)	4.27 (2.83)	2.00 (0.89)^#†^	< 0.001
HOMA-IR ^a^	− 0.07 (1.07)	0.85 (1.20)*	0.88 (1.01)^#^	< 0.001	0.32 (0.57)	0.76 (0.66)*	1.56 (0.50)^#†^	< 0.001
Insulin secretion ^b^	0.48 (0.37)	0.53 (0.25)	0.66 (0.41)^#^	0.008	0.41 (0.28)	0.44 (0.21)	0.70 (0.53)^#†^	0.003

The data are mean (SD).

*AUC* area under the curve, *HDL-C* high-density lipoprotein cholesterol, *LDL-C* low-density lipoprotein cholesterol.

^a^log-transformed.

^b^Insulin secretion (InsAUC30/GluAUC30); the statistical test conducted using ANOVA.

*Compare between NOH and MHO, p-value < 0.05.

^#^Compared between NOH and MUHO, p-value < 0.05.

^†^Compared between MHO and MUHO, p value < 0.05.

Glucose-related cardiometabolic traits also showed differential levels between participants without obesity and with obesity, and the differences were mainly shown between NOH and MUHO groups (Table 2). Fasting glucose levels had significant higher levels in MUHO compared to NOH groups (Fig. 1B) and were only show higher levels in MHO compared to NOH groups among participants at baseline (Table 2 and SI Fig. S2). For HbA1c levels, no significant differences were observed among obesity groups in the baseline, but HbA1c levels had an increasing trend (p-value = 0.003 in linear trend) and show significant differences in the follow-up (SI Fig. S2B). For two glucose-related traits in the OGTT including glucose AUC 2-h and glucose 2-h levels, significant elevations in glucose AUC 2-h were observed between MUHO and NOH. Glucose tolerance and AUC 2-h did not show significant differences comparing MHO and MUHO groups and comparing MHO and MUHO in the baseline (Fig. 1B), but showing deteriorating trend (p-value < 0.001 in linear trend). In the follow-up, we observed significant differences between MHO and MUHO for glucose AUC 2-h (SI Fig. S2B).

Insulin-related traits showed significant differences between NOH and MUHO groups (Fig. 1C). Fasting insulin significantly elevated among MHO and MUHO participants compared to NOH in two visits (Table 2 and SI Fig. 1C). Insulin resistance (HOMA-IR) showed significant increasing trend and insulin sensitivity (Matsuda index) shown significant deceasing trend among three groups of NOH, MHO and MUHO in both baseline and follow up visits (p-value < 0.001 in linear trend) (Fig. 1C and SI Fig. S2C). Additionally, insulin secretion (InsAUC30/GluAUC30) was higher in MUHO compared to NOH group, while no significant differences were observed between MHO and NOH groups.

### Proteomic signatures associated with obesity phenotypes and cardiometabolic traits

**Figure 2:**
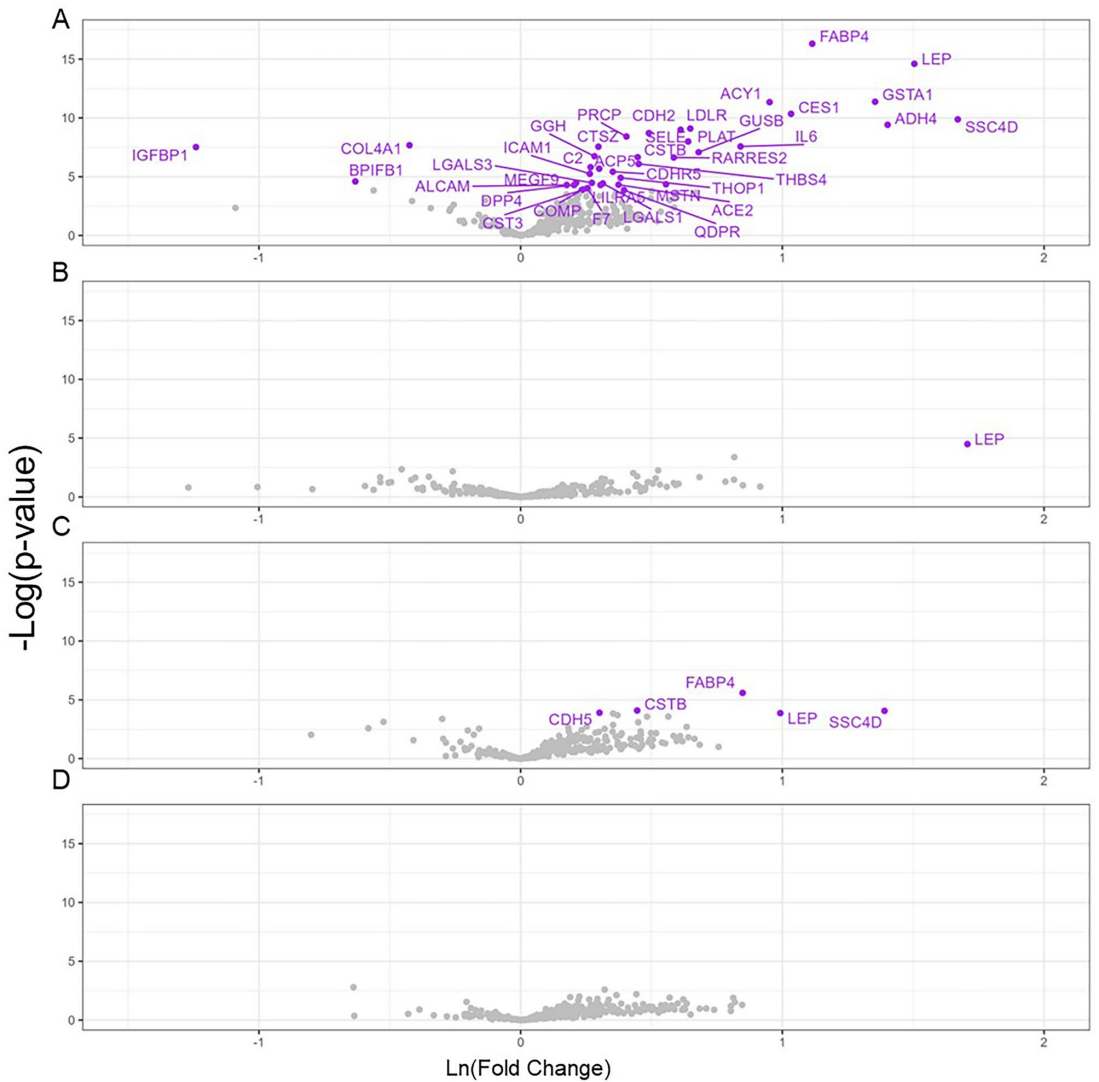
Proteins Differentially Expressed Among Obesity Groups at Baseline and Follow-up Visit. (**A**) Comparing participants with obesity (MHO and MUHO, N = 61) and without Obesity (N = 95) at baseline; (**B**) Comparing participants with MHO (N = 8) and without Obesity (N = 95) at baseline; (**C**) Comparing participants with obesity (MHO and MUHO, N = 40) and without Obesity (N = 40) at follow up; (**D**) Comparing participants with MHO (N = 13) and without Obesity (N = 40) at follow up; MHO: metabolically health obesity, MUHO: metabolically unhealthy obesity, NOH: non-obesity healthy.

In the baseline, a total of 346 proteomic markers passed QC from 156 participants were used to assess the differentially expressed proteins between MHO (N = 8) and NOH (N = 95) and between participants with obesity (MHO and MUHO, N = 61) and NOH (N = 95). After FDR correction, we found that 1 (overexpressed) and 39 (36 overexpressed and 3 underexpressed) protein markers were differentially expressed when comparing the MHO to the NOH group and when comparing the obesity with the NOH groups at baseline, respectively (Fig. 2). Among these proteins, leptin (*LEP*) levels were significantly overexpressed in both comparisons between MHO with NOH and comparison between obesity with NOH groups. Three protein markers: *IGFBP1*, *BPIFB1* and *COL4A1* were significantly underexpressed, comparing between participants with obesity and NOH group. Similar results were found at the follow up visit for a subset (N = 80) of participants, and five proteins were elevated in the obesity group compared to NOH group, four of which (*LEP*, *CSTB*, *FABP4* and *SSCAD*) were also identified in the baseline. However, no significantly expressed proteins were found between MHO and NOH groups at follow up. In addition, we did not identify any significantly expressed proteins between MHO and MUHO in both baseline and follow-up visits, with FDR < 0.05. We only found that 21 and 16 proteins differentially expressed between MHO and MUHO with p-value < 0.05 in the baseline and follow-up visits, respectively (SI Table S3). Among them, 9 proteins overlapped with differentially expressed proteins between obesity and NOH. Specifically, *BPIFB1* underexpressed and *ADH4, CSTB, FABP4, GGH, LEP, GUSB, LGALS1, MEGF9* and *SIRPA* proteins overexpressed in MUHO vs MHO groups.

**Figure 3:**
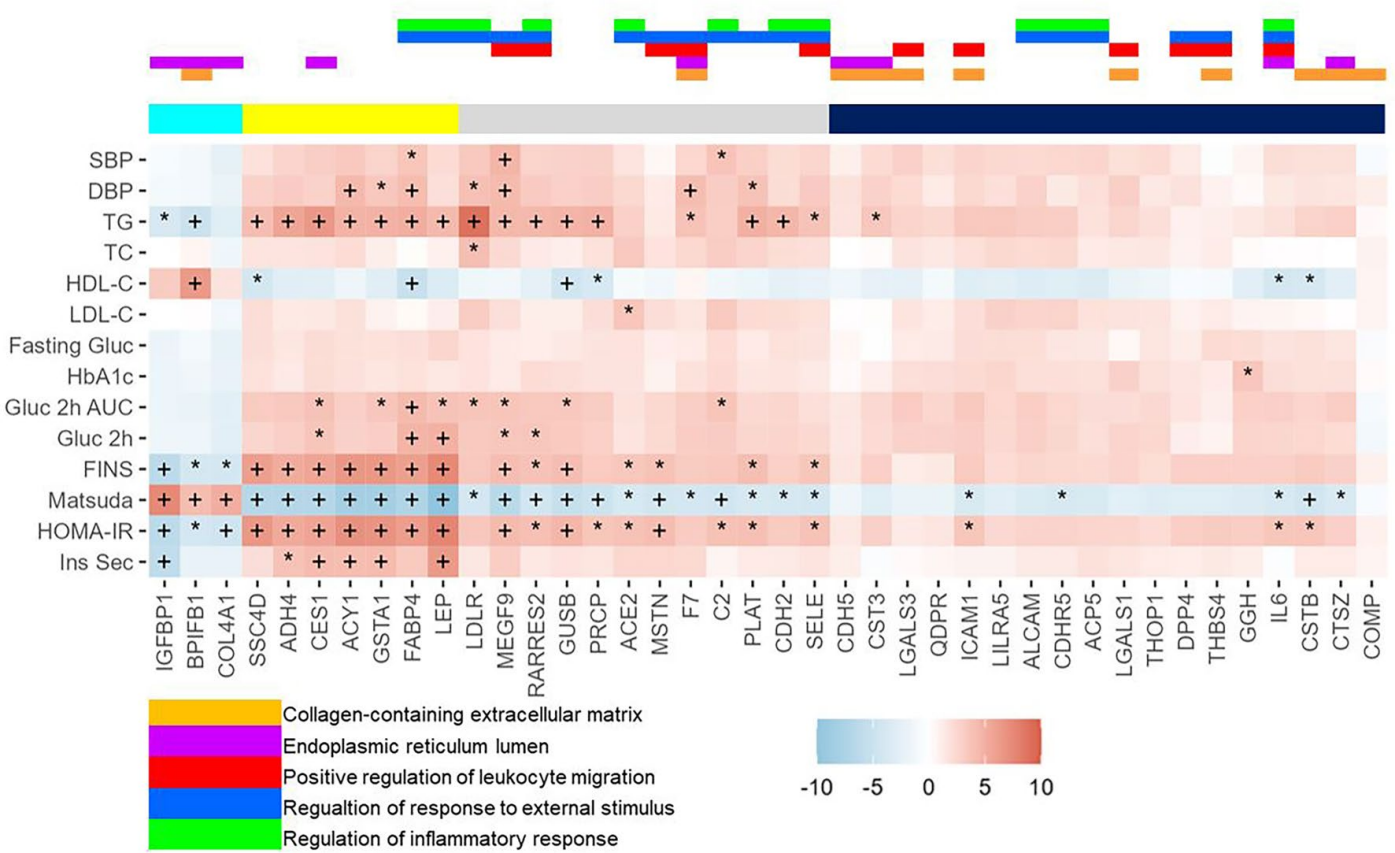
Protein Differentially Expressed with Cardiometabolic Traits in Longitudinal Analysis. Heat map of associations with 40 obesity group differentially expressed proteins and main cardiometabolic traits with hierarchical clustering of proteins. The color of heat map shows the t-score of the GEE model, red indicates positive associations and blue indicates negative associations. The color bar on the top indicated the overlapping GO terms. *FDR < 0.05, ^+^FDR < 0.005.

The longitudinal association between 40 differentially expressed proteins and cardiometabolic traits of lipid profiles, glucose- and insulin-related traits and blood pressure are shown in Fig. 3. This figure shows that these 40 differentially expressed proteins were primarily associated with cardiometabolic traits of lipids including triglycerides and insulin profiles such as fasting insulin, insulin resistance (HOMA-IR) and insulin sensitivity (Matsuda index). We found that three protein markers that were decreased with obesity group (*IGGBP1*, *COL4A1* and *BPFB1*) also had consistent associations with cardiometabolic traits, which were in inverse direction compared to other protein markers and clustered in one cluster (turquoise) in hierarchical clustering. Another two clusters (yellow and grey) included 7 and 12 proteins with significant associations with cardiometabolic traits, respectively. Last cluster (black) contains 18 proteins, which did not show strong associations with the traits. SI Fig. S3 in the supplementary material shows the cross-sectional associations between differentially expressed proteins with cardiometabolic traits at the baseline and follow up visits separately. The cross-sectional associations support the findings of longitudinal analysis that triglycerides and insulin-related traits were mostly strongly associated with proteins.

### Enriched biological pathways shown by differentially expressed proteins by obesity groups

**Figure 4:**
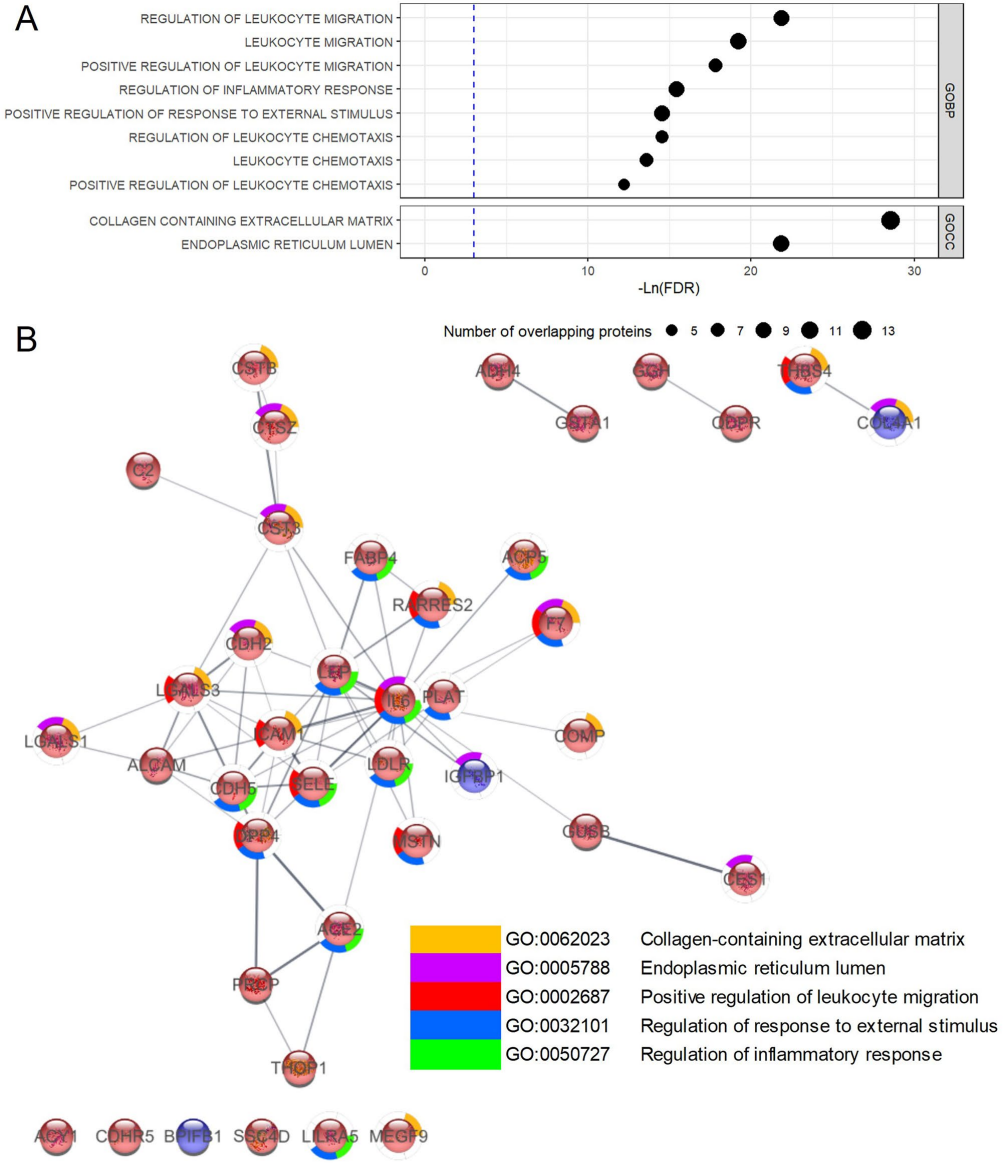
Overrepresentation Analysis (ORA) of Gene Ontology (GO) Terms for 40 Differentially Expressed Proteins Across Obesity Groups (**A**) and STRING Protein–protein Interaction Network (**B**). (**A**) Blue line indicated the FDR = 0.05, size of the point indicates the number of overlapping proteins in each GO terms, (**B**) Red and blue fill color of protein indicates overexpression/underexpression of protein with obesity groups, the color of outer circle of the protein indicates the overlapping GO terms for each protein. The edge between proteins indicates there are either known interaction, predicted interaction, co-expression, protein homology or from text-mining between two proteins. *GOBP* Gene Ontology Biological Process, *GOCC* Gene Ontology Cellular Composition.

The over representation analysis (ORA) based on GO terms found enrichments in multiple networks for 41 differential expressed proteins with obesity (Fig. 4A). Among top 10 enriched GO terms, 8 were GO biological process terms and 2 GO cellular composition terms, all with FDR < 0.005. These highly enriched terms were mainly related to immune and inflammation (regulation of leukocyte migration, inflammatory response, and response to external stimulus), collagen containing extracellular matrix and endoplasmic reticulum (ER) function. STRING protein–protein interaction (PPI) network analysis identified 1 inter-connected network of 28 proteins, and the overlapped proteins in the most enriched GO terms were primarily found in the PPI network (Fig. 4B). Proteins (*IGBFP1*, *BPIFB1* and *COL4A1*) with negative association with obesity and cardiometabolic traits (turquoise cluster in Fig. 3) all overlapped in ER lumen GO term. Proteins in grey clusters overlapped mainly in regulation of response to external stimulus and regulation of inflammatory response, and most proteins in yellow cluster did not overlap top GO terms. We found six proteins (*SSC4D, ADHD4*, *ACY1*, *GSTA1*, *GUSB* and *PRCP*) that did not overlap with top enriched pathways also showed strong associations with cardiometabolic traits including insulin-related traits and triglycerides (Fig. 3). The proteins in dark blue cluster showed moderate overlapping with GO terms but did not show strong associations with cardiometabolic traits.

Likewise GO ORA, the IPA found similar results and identified 45 diseases and biofunctions that were significantly enriched (SI Table S2), most of which were shown to be activated (40 activated and 5 inhibited). The top 5 enriched bioprocess were vasculogenesis, development of vasculature, migration of cell, migration of phagocytes and inflammatory responses and 27 proteins overlapped in these processes (SI Fig. S4) which were largely in the PPI network identified by STRING. The enriched diseases and biofunctions of IPA were related to cellular movement, immune cell trafficking, tissue development and inflammatory response (SI Fig. S5). Additionally, IPA found 55 canonical pathways were significant enriched, but only 1 pathway (HMGB1 Signaling) passed |Z-score|> 2 cut-off value (SI Table S3). Sensitivity analysis supports the robustness of ORA. Since proteins from the Cardiometabolic panel were pre-selected, by using all proteins from the Olink Cardiometabolic Panel as background, we still found five GO terms related to inflammation (regulation of response to external stimulus, regulation of leukocyte migration, regulation of inflammatory response and regulation of defense response) and ER function (ER lumen) were also enriched with FDR < 0.2 (SI Fig. S6).

## Discussion

In this study, we assessed cardiometabolic traits and plasma proteomics that were associated with obesity among a cohort of young adults with a history of having childhood obesity. We found that obesity and MHO were associated with deteriorated cardiometabolic traits including lipids profiles and insulin resistance compared to NOH. Among cardiometabolic traits, TC and insulin-related traits including insulin sensitivity, insulin resistance and insulin secretion were discriminant between MHO and NOH groups. In addition, we identified 40 proteins significantly differentially expressed with obesity, most of which were also shown strong associations with insulin-related traits and lipid profiles. Among these proteins, 37 were overexpressed and 3 were underexpressed. These differentially expressed proteins implied perturbation of pathways of inflammation, extracellular matrix remodeling and ER functions among a population with obesity.

Although previous studies pointed out that populations with MHO could be protected from several obesity-related cardiometabolic disorders, such as cardiovascular diseases, diabetes or cancer^6,15,16^, other studies also reported that MHO was also associated with higher risk^7,17^. In our study, even NOH group is defined to have up to 1 impaired metabolic traits, while MHO group is with no impaired traits^18^, we also noticed that deteriorated cardiometabolic health such as lipid profiles and insulin sensitivity between MHO and NOH (Fig. 1 and Table 2). This indicates that a population with obesity that has normal levels of blood pressure, HDL-C, triglycerides, and fasting glucose, also exhibits hyperinsulinemia, dysregulated glucose metabolism, and insulin resistance shown in OGTT. Our results are consistent with previous studies that MHO was not protective from cardiometabolic diseases, that obesity could lead to impaired beta-cell function and dysregulated insulin homeostasis compared to normal weight population^5,19–21^. The results from our study supports that MHO definition including insulin sensitivity could better indicate disease outcomes or mortality as suggested by previous studies^6,17^.

We identified 37 proteins that were over expressed, and 3 proteins were underexpressed with obesity comparing to non-obesity, respectively. Most of the these differentially expressed proteins were found in the baseline, while we also found 5 proteins differentially expressed in the follow-up. Among these 40 proteins, 29 proteins showed significant associations with at least 1 cardiometabolic traits, most of which were clustered in the turquoise, yellow and grey clusters in the hierarchical clustering. Three proteins underexpressed with obesity (*IGFBP1*, *BPIFB1* and *COL4A1*) were clustered in the turquoise cluster and overlapped with ER lumen and collagen-containing extracellular matrix GO terms. These three proteins were strongly associated with insulin traits. Previous studies have also found insulin growth factor binding protein 1 (*IGFBP1*) showing protective effects for diabetes^22^ and metabolic syndrome^23^. Similar to turquoise cluster, proteins in the yellow cluster also showed strong association with insulin traits and triglycerides. Interestingly, 4 proteins (*SSC4D*, *ADH4*, *ACY1* and *GSTA1*) in this cluster did not overlap with top GO terms but showed strong associations with cardiometabolic traits. *ACY1* (Aminoacylase 1) and *ADH4* (Alcohol dehydrogenase 4) both are enzymes involved in the amino acid and carbohydrate metabolism pathways. *ACY1* has been shown previously related to type 2 diabetes risk in a large longitudinal cohort and shown associations with insulin homeostasis in vitro and in vivo^24^. *ADH4* is an enzyme metabolizing ethanol and has also been shown to be positively related to metabolic disorder such as non-alcoholic steatohepatitis^25^. Additionally, three proteins (*LDLR*, *MEGF9*, *C2*) in the grey cluster mostly overlapped with inflammation pathways of GO term and showed strong associations with obesity, insulin resistance and blood pressure. It is believed that inflammation is a major component of obesity^12,26,27^ and it has been associated with multiple obesity-related cardiometabolic traits including insulin resistance^28^, impaired glucose tolerance^29^ and dyslipidemia^30^. In this study, we identified several proteins (*FABP4, LEP, RARRES2, MSTN, C2, SELE* and *IL6*) that belong to a family of chronic pro-inflammatory cytokines and are primarily produced in response to infection or stress, some of which are mainly produced by adipose tissue (*FABP4, LEP* and *RARRES2*). Four proteins in the grey cluster (*PRCP, ACE2, F7,* and *PLAT*) that modulate vasoconstriction or coagulation could be triggered by obesity-induced endothelial cell injury^31^. The rest of the protein markers that were significantly associated with cardiometabolic traits, mainly in dark blue cluster (*ICAM1, CDHR5, DPP4, CSTB* and *CTSZ*), play roles in cell adhesion, extracellular matrix remodeling and are involved in ER function. Recently, studies have found that extracellular matrix remodeling could be an important mechanism mediating obesity and cardiometabolic outcomes such as insulin resistance^32,33^, metabolic syndrome^34^ and type 2 diabetes^35^, through generating (1) mechanical barriers for glucose and insulin transporting to liver and muscle, and (2) alternating integrin signaling that culminate in impaired insulin action^33^. The plasma proteins that are significantly associated with obesity are secreted in different tissues or organs such as in adipose tissue (*LEP*, *FABP4*) or liver (*ADH4*, *IGFBP1*), these findings may further the understanding mechanisms linking obesity and impaired metabolic health with a focus on organ cross-talk^36–38^. Other proteomic studies on obesity groups found similar results. One larger study of over 1000 participants in Germany and Qatar found plasma *LEP* and *IGFBP1* causally associated with BMI and obesity^39^. Another study of 150 participants (100 with obesity and 50 without obesity) in the Netherlands found that *SELE*, *IGFBP1* and *RARRES2* were significantly different between participants with obesity versus without obesity^40^. Our findings of inflammatory proteins related to obesity are supported by studies in Sweden^41^ and Qatar^42^, that inflammatory proteins were significantly overexpressed with obesity, especially in immune response, chemokine signaling and tissue repair, and remodeling pathways. Despite the limited sample sizes of MHO phenotype, we also found MHO differed from MUHO in several cardiometabolic traits including HDL at baseline (Fig. 1) and differed in fasting glucose, glucose levels at 2 h, Matsuda index, HOMA-IR and insulin secretion in OGTT at follow-up (SI Fig. S2). In addition, among 40 differentially expressed proteins between obesity and NOH, 9 proteins differentially expressed between MHO and MUHO at p-value < 0.05 (SI Table S3). These differences between MUHO and MHO might explain the MHO is a transient phenotype that has more protected glucose metabolism and insulin sensitivities, as indicated by previous studies^43^.

There are a few limitations of our study. Firstly, due to the limited numbers of MHO phenotype in this cohort, we only found leptin *(LEP)* differentially expressed (FDR < 0.05) between MHO and NOH group in the baseline. The small samples of MHO limited the power to detect proteins that were significantly differentlialy expressed between MHO and NOH in the follow-up visit, and between MHO and MUHO, respectively. We also did not find significant differences in abdominal fat between MHO and MUHO (Table 1), even though abdominal fat is believed to be a better predictor of cardiometabolic disease than BMI^2,16^. We also only used the Olink Cardiometabolic Explore Panel, which has limited coverages of inflammatory interleukins and chemokines. In addition, the results of this study are from a young population and may not be generalizable to other populations without an independent replication. The major strength of this study is that we combined extensive phenotyping including anthropometry, OGTT and detailed dietary recall with plasma proteins, to assess cardiometabolic traits and molecular mechanisms differed by obesity. Another advantage of this study is that we used a longitudinal design with 4.6 years follow-up compared to previous studies^44^.

In summary, using targeted Olink Cardiometabolic Explore proteomics of 384 plasma proteins in 156 young adults, we identified 40 proteins associated with obesity phenotypes compared to non-obesity and 29 of which were related to obesity-related cardiometabolic traits including insulin resistance and dyslipidemia. Among them, only leptin *(LEP)* was found to be differentially expressed between MHO and MUHO. The significantly differentially expressed proteins represented a diverse physiological process such as inflammation and immunomodulation, extracellular matrix remodeling and ER function, reflecting the obesity progression and subsequent diminished adverse cardiometabolic outcomes. The findings of this study provided complementary information to improve understanding of obesity, and highlighted applications of plasma proteomics as potential biomarkers of obesity-related cardiometabolic disorder.

## Methods

### Study participants

In this longitudinal Meta-AIR study, we recruited 172 participants between age 17–22 years during 2014–2018, from the Children’s Health Study (CHS)^45,46^. Study participants were selected if (1) they had overweight or obesity history in early adolescence and (2) they had not been diagnosed with diabetes, had no medical conditions and were not taking medications that affect glucose metabolism. During the baseline visit, we conducted detailed questionnaire interviews, anthropometry, fasting blood collection, oral glucose tolerance tests (OGTT) and 24-h dietary recalls^47^. At baseline, 156 of the participants were included in this study with complete data. Between 2020 and 2022, among 156 participants 82 completed follow-up visits, with an average 4.6 years. Participants in the follow-up visits underwent the same procedures as baseline visits. Both baseline and follow-up visits were conducted at University of Southern California (USC) Diabetes and Obesity Research Institute. Due to the nature of this study which is based on existing cohort resource (Meta-AIR cohort in CHS), we have not calculated sample size prior to the visit. SI Fig. S7 shows the number of participants included in the baseline and follow-up visits and excluded in the study. This study was approved by the USC Institutional Review Board (IRB # HS-19-00338), written informed consent was obtained from all participants and all study procedures were performed in accordance with relevant guidelines and regulations.

### Physical examinations and questionnaires

In both baseline and follow-up visit, we conducted physical examinations on each participant prior to blood collection and OGTT. In the physical examinations, we conducted anthropometry measurements including measurement of height, weight and waist circumference. Body fat percent was measured using dual-energy X-ray absorptiometry scan and abdominal MRI measures adiposity (subcutaneous abdominal adipose tissues (SAAT), visceral adipose tissue (VAT) and hepatic fat faction (HFF)) were conducted. Obesity was defined as BMI >= 30 kg/m^2^. In addition, we measured systolic blood pressure (SBP), diastolic blood pressure (DBP) and heart rate using an automatic sphygmomanometer on the left arm in the siting position. Detailed questionnaires collecting sociodemographic, smoking and medical history and 24-h dietary recalls were administered at baseline and follow-up visits, based on which we estimated Dietary Approaches to Stop Hypertension (DASH) index^48^.

### Oral glucose tolerance test (OGTT)

All participants underwent an OGTT (0, 30, 60, 90, 120 min) in the morning after fast using a load of 75 g anhydrous glucose dissolved in water. Participants were instructed to maintain a standard diet for at least 3 days before the tests. Blood samples were collected at 0, 30, 60, 90 and 120 min after the glucose challenge for measurement of glucose and insulin. Fasting glucose (FGlu) and fasting insulin (FINS), and total areas under the curve (AUC) at 2-h of glucose (GluAUC_120_) and insulin (InsAUC_120_) from OGTT were calculated using the trapezoidal method. Insulin sensitivity was measured by the Matsuda index from OGTT test as $\frac{1000}{\sqrt{{\text{FGlu}}*{\text{FINS}}*{{\text{GluAUC}}}_{120}*{{\text{InsAUC}}}_{120}}}$^47^. We used InsAUC_30_/GluAUC_30_, the ratio of insulin and glucose area under the curve for first 30 min of the OGTT to represent the main insulin secretion index^19^. In addition, we calculated homeostatic model assessment for insulin resistance (HOMA-IR), an index which estimates insulin resistance, using the equation FGlu * FINS/405.

### Serum lipids and plasma proteomics profiling

Fasting serum samples were collected before the OGTT test using 4 mL serum separator tube. Serum lipid samples were stored at − 80 °C and later assayed in duplicate by Fujifilm Wako Diagnostics enzymatic assay^47^. In the baseline and follow-up visit, triglycerides, total cholesterol (TC), high-density lipoprotein-cholesterol (HDL-C), low-density lipoprotein-cholesterol (LDL-C) were measured in serum samples. Fasting plasma samples were collected at both baseline and follow-up and stored at − 80 °C. EDTA plasma samples collected at the baseline visit were shipped with dry ice to the Olink Inc. laboratory in Waltham, MA USA, and 50 µL of samples were used for the proteomic analysis. We used Olink Explore 384 Cardiometabolic panel, a high-throughput proximity extension assay (PEA) in combination with next generation sequencing readout, to profile proteomic features in plasma samples^49^. The Cardiometabolic Explore Panel covers plasma proteins of interest for cardiovascular and metabolic diseases (Table S1 for protein list) and gives the normalized protein expression (NPX) as a log_2_-transformed relative protein concentration. Two plates were used with all samples randomized. Inter-plate normalization was conducted using bridging reference samples before generating final the NPX value. Limits of detection (LOD) of each protein were determined by negative controls. Sample protein NPX values below LOD were replaced by LOD, and 25 proteins were removed from the sample since more than 25% of all samples were below the LOD, resulting in 346 protein features for baseline visits consisted of 169 samples.

### Metabolically healthy obesity (MHO)

Based on the National Cholesterol Education Program (NCEP) Adult Treatment Panel III (ATP-III) criteria^18^, we defined the metabolically healthy obesity (MHO) using the strict definition without waist circumference as used more recently^2,6,50^: (1) participants need to have BMI >  = 30 kg/m^2^, (2) have none of the following metabolic criteria: serum triglycerides > 150 mg/dL (1.7 mmol/L), HDL-C serum concentrations < 38.6 mg/dL (1.0 mmol/L) for male or < 50.3 mg/dL (1.3 mmol/L) for female, SBP > 130 mmHg or DBP > 85 mmHg or taking anti-hypertensive treatment, and fasting blood glucose > 100 mg/dL (6.1 mmol/L) or taking diabetes treatment. For participants with BMI over 30 kg/m^2^ and satisfied one or more above metabolic criteria, they were categorized as metabolically unhealthy obesity (MUHO) at that study visit. For participants without obesity, we characterized them as non-obesity heathy (NOH) if they met no more than one of above metabolic criteria. Fourteen participants (N = 12 in the baseline and N = 2 in the follow-up) without obesity met more than one metabolic criteria, therefore, were removed in the analysis.

### Statistical analysis

Baseline population demographics by obesity group (NOH, MOH and MUHO) were tested using Fisher’s exact test for categorical characteristics and Wilcoxon rank sum test for continuous outcomes. Cardiometabolic continuous traits including lipid profiles (triglycerides, TC, HDL-C, LDL-C), glucose metabolism (fasting glucose, HbA1C, GluAUC_120_ and glucose-2 h) and insulin traits (FINS, Matsuda index/insulin sensitivity, HOMA-IR/insulin resistance, and insulin secretion) that were associated with obesity groups were assessed using one-way ANOVA, separately at baseline and follow-up visits, as well as combined. Trend tests were conducted to assess whether cardiometabolic traits showed trends across NOH, MHO and MUHO groups at baseline and follow-up separately. Raw two-sided p-values < 0.05 were used as a statistically significant cut-off value. Then, the log2 transformed protein expression levels (NPX) were used as dependent variables and obesity groups were used as independent variables in multivariate linear model, to assess associations between NPX and obesity. We controlled for sex, age, ethnicity, parental education level, smoking status and DASH nutrition index in the analysis. We used Benjamini-Hochberg (BH) procedure to calculate the false discovery rate (FDR) accounting for multiple testing of number of proteins (N = 346), with FDR < 0.05 as cut-off^51^. Lastly, we aim to understand how obesity-related proteins were related to cardiometabolic traits. By using marginal linear models with generalized estimating equations (GEE), we assessed the associations between differentially expressed proteins and cardiometabolic traits of lipid profiles, glucose- and insulin-related traits both at baseline and follow-up. Hierarchical clustering was conducted to further cluster significant proteins. Same covariates were adjusted, and BH procedure was used to calculate the FDR considering both number of proteins and cardiometabolic traits. R software (version 4.1.3) was used in the statistical analysis.

### Enriched protein pathway analysis

For proteins that were differentially expressed between obesity groups, we conducted over-representation analysis (ORA) based on gene set annotations from Gene Ontology (GO) using *ClusterProfiler* R package^52^. The Fisher Exact Test (FET) was used to determine significantly enriched GO terms based on FDR < 0.05, with all proteins used as background. Then, we used STRING database (version 11.5) to assess protein–protein interaction (PPI) for differentially expressed proteins^53^, and examined the PPI network among the differentially expressed proteins. Additionally, we leveraged Ingenuity Pathway Analysis platform (IPA, Qiagen Inc.) to conduct ORA for canonical pathways and diseases and biofunctions curated from Qiagen Knowledge Base^54^. We also assessed the activation/inhibition patterns of the canonical pathways and disease and biofunctions, based on Z-score from the IPA, a statistics showing how well the match between overexpressed/underexpressed protein patterns and the predicted activation/inhibition pattern of overlapping network^55^. In IPA, we also used FDR < 0.05 in FET in enrichment analysis and |Z-score|> 2 as cut-offs. Lastly, to assess the robustness of the ORA results, we conducted the sensitivity analysis using all 384 proteins of the Olink Cardiometabolic Explore panel as background in ORA.

### Supplementary Information

Supplementary Figures.

Supplementary Tables.

## Data Availability

The datasets generated during the current study are available from the corresponding author on reasonable request.
