# Supplementary material for: Cardiometabolic profiles and proteomics associated with obesity phenotypes in a longitudinal cohort of young adults
Source: Sci Rep. 2024 Mar 28;14:7384. doi: 10.1038/s41598-024-57751-2 (PMC10978904; doi:10.1038/s41598-024-57751-2)

Supplementary Information for

Cardiometabolic Profiles and Proteomics Associated with Obesity Phenotypes in a Longitudinal Cohort of Young Adults

Table of Contents

Figure S1: Sankey Diagram Showing the Change of NOH, MHO and MUHO Group Status for N = 80 Participants with Two Visits at Baseline and Follow-up.

Figure S2: Protein Differentially Expressed Among Obesity Groups at Baseline and Follow-up Visit.

Figure S3: Heat map of associations among 40 serum proteins and main cardiometabolic traits in the baseline visit (A) and follow-up visit (B)

Figure S4: Top activated networks of biofunctions and diseases and 27 overlapping proteins for differentially expressed proteins in IPA

Figure S5: Categories of Diseases and Biofunctions Enriched from IPA

Figure S6: GO Terms adjusting for Olink as Background

**Figure S1. Sankey Diagram Showing the Change of NOH, MHO and MUHO Group Status for N = 80 Participants with Two Visits at Baseline and Follow-up.** NOH: non-obesity healthy, MHO: metabolically healthy obesity; MUHO: metabolically unhealthy obesity.


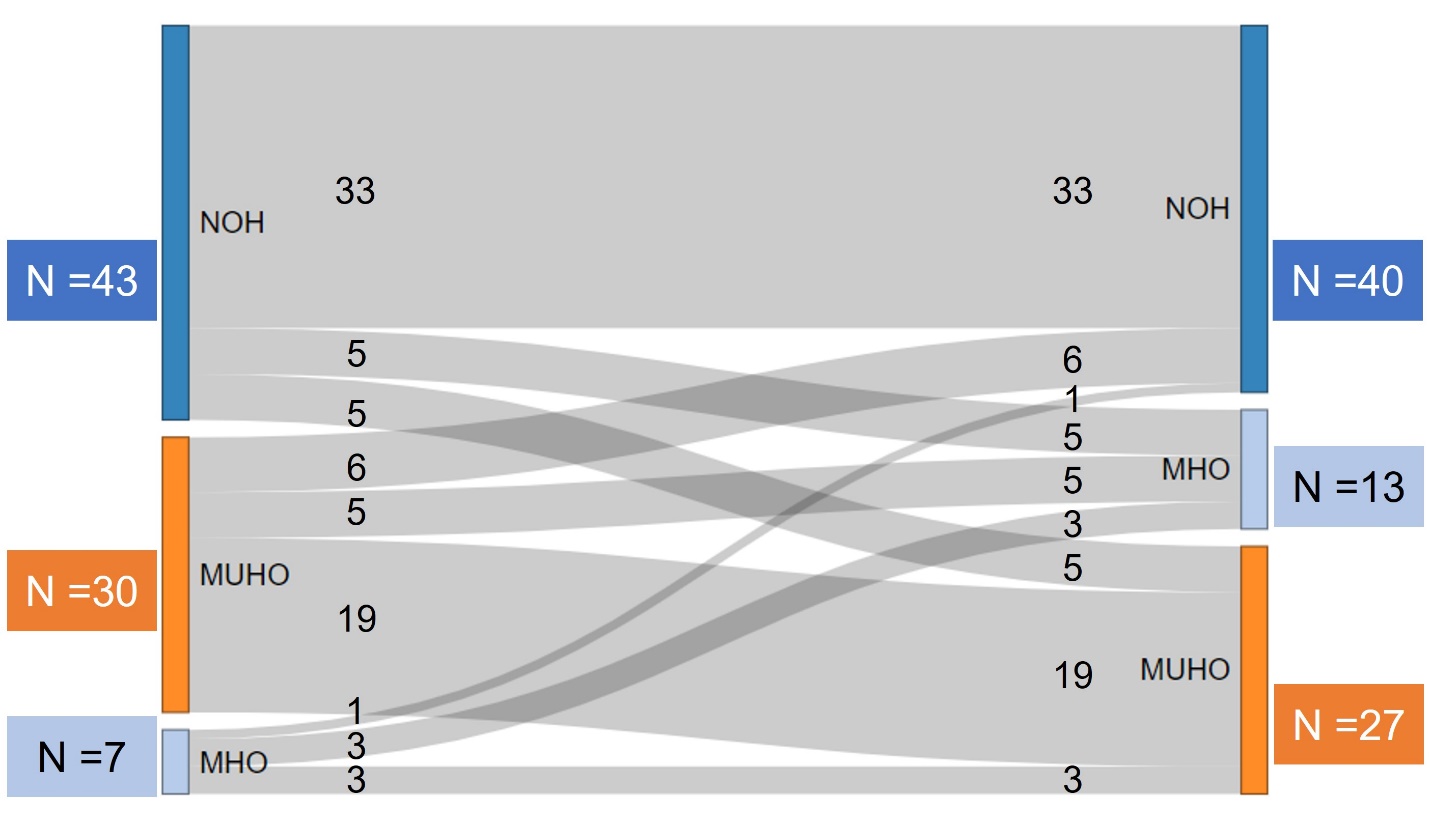


**Figure S2. Characteristics of cardiometabolic traits by non-obesity healthy (NOH), metabolically healthy obesity (MHO) and metabolically unhealthy obesity (MUHO) groups in follow-up.** A: lipid profiles including triglycerides, total cholesterol, HDL-cholesterol and LDL-cholesterol. B: glucose metabolism related outcomes including fasting glucose levels, HbA1C, 2-hour glucose under the curve (AUC) in OGTT and 2-hour glucose levels in OGTT. C: insulin related outcomes including fasting insulin, insulin sensitivity (Matsuda index), insulin resistance (HOMA-IR) and insulin secretion (InsAUC30/GluAUC30) in OGTT.


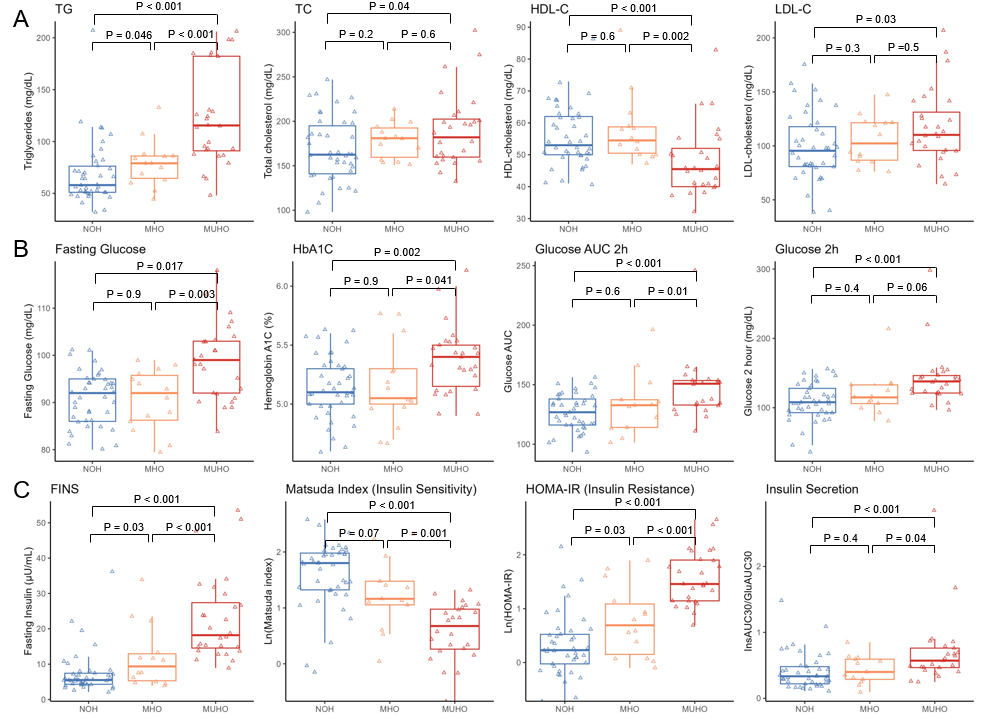


**Figure S3. Heat map of associations among 40 serum proteins and main cardiometabolic traits in the baseline visit (A) and follow-up visit (B).** The color of heat map shows the t-score of the linear regression model, red indicates positive associations and blue indicates negative associations. * FDR < 0.05, + FDR < 0.005.


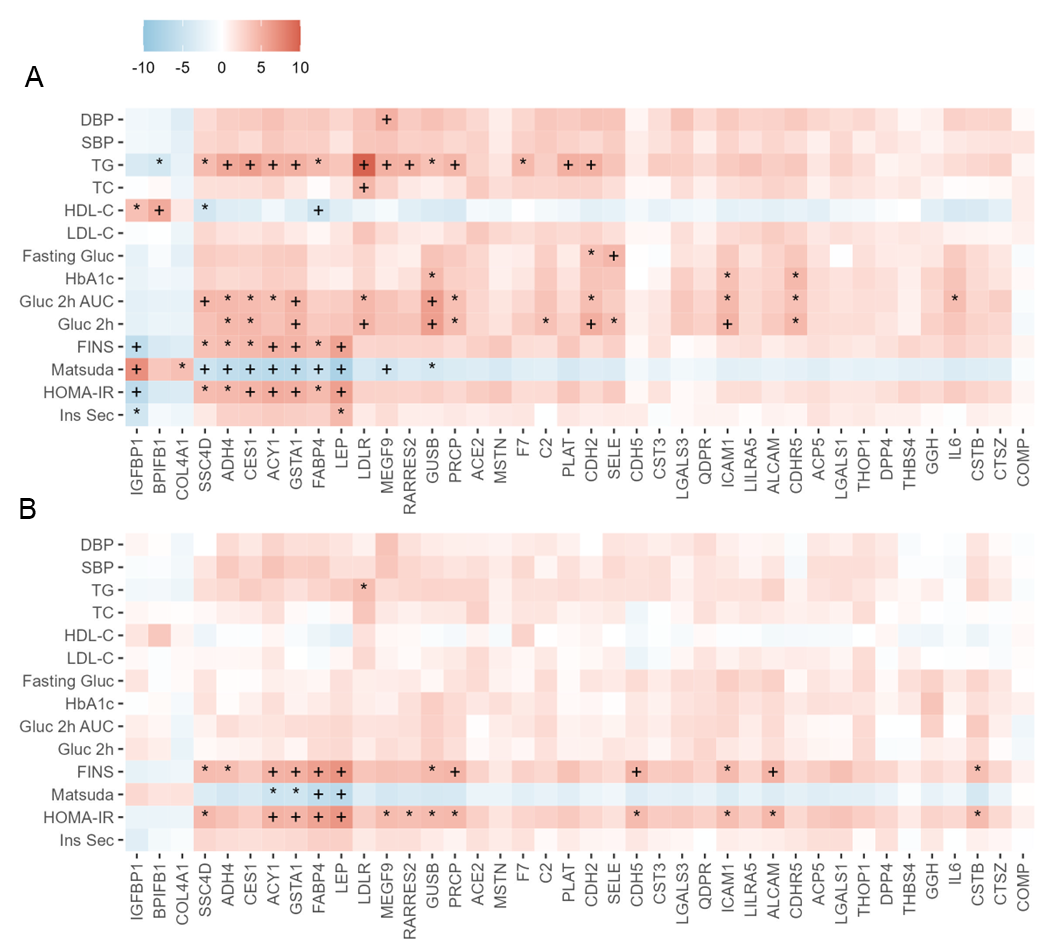


**Figure S4. Top activated networks of biofunctions and diseases and 27 overlapping proteins for differentially expressed proteins comparing obesity vs. non-obesity in IPA.** The color of the protein indicated overexpressed (red) or underexpressed (green), and color of the line indicated proteins activate (orange) or suppress (yellow) each function.


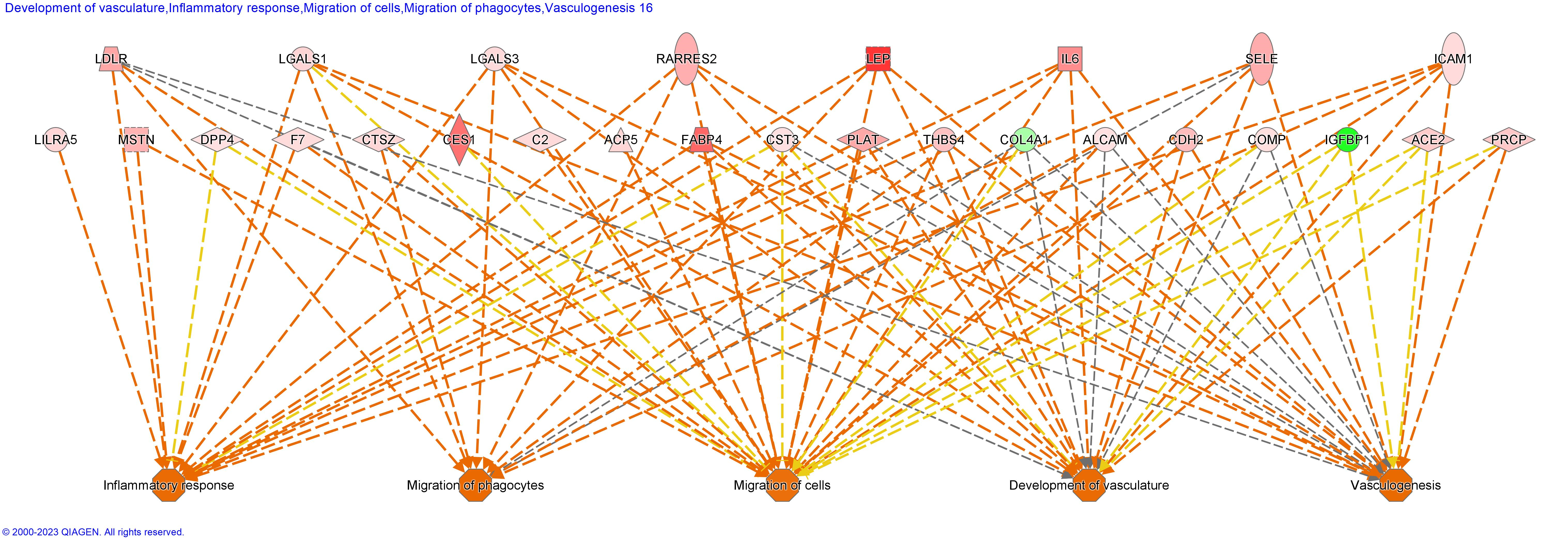


**Figure S5. Categories of Diseases and Biofunctions Enriched from IPA.** The x-axis shows the -log(p-value) of the diseases and biofunctions enriched with 40 proteins from IPA.


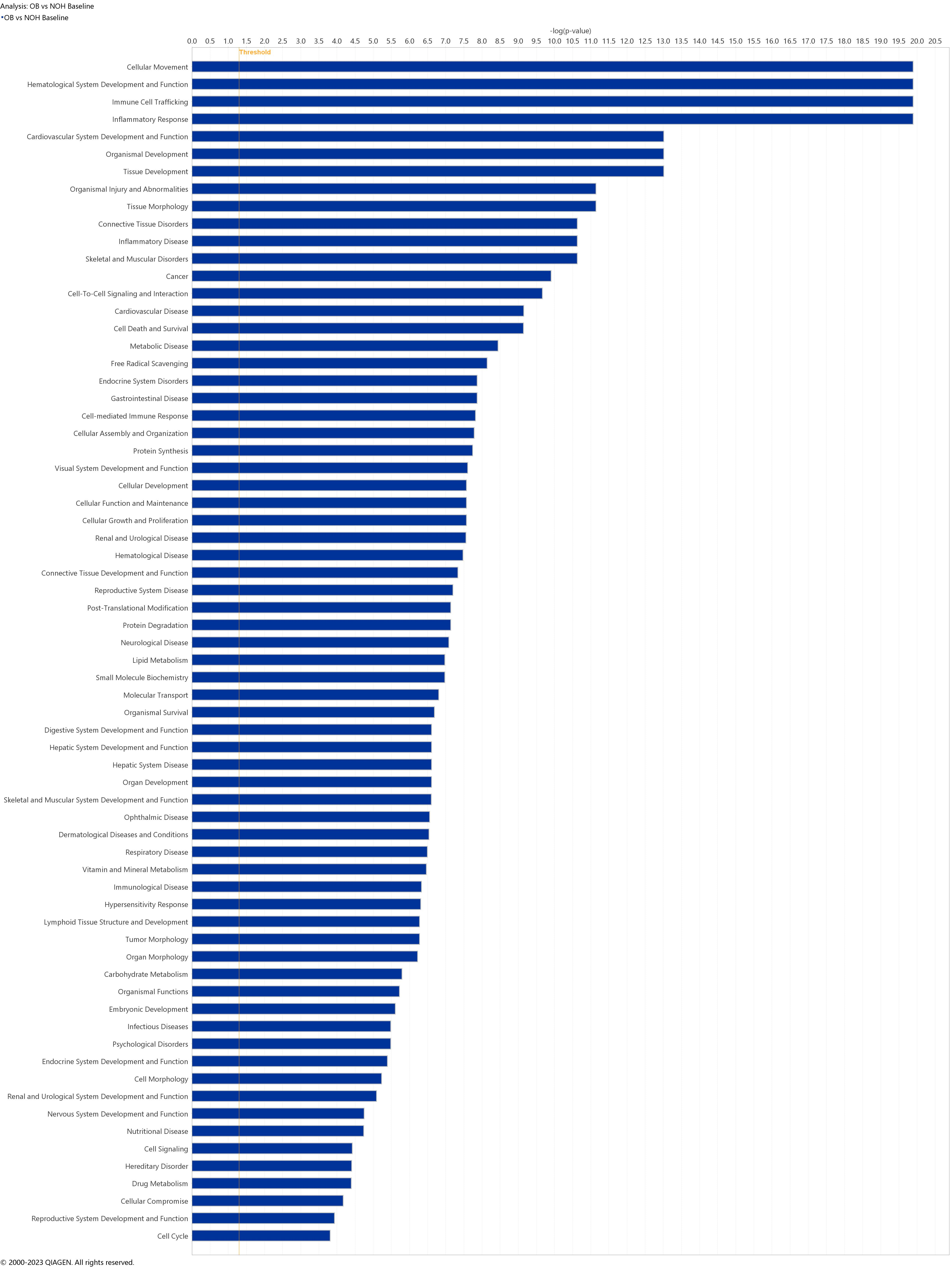


**Figure S6. GO Terms Adjusting for Olink Cardiometabolic Proteins as Background.** Blue lines indicated FDR < 0.0 and purple lines indicated FDR < 0.2.


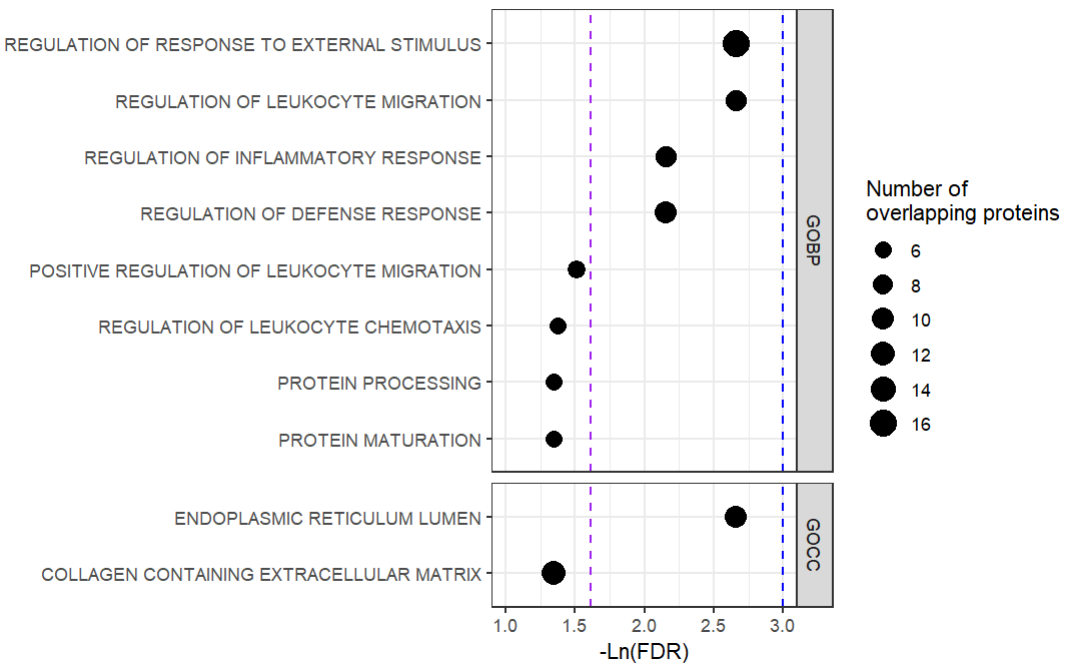


**Figure S7. Flow Chart Shoring the Number of Participants in the Baseline and Follow-up Visits**


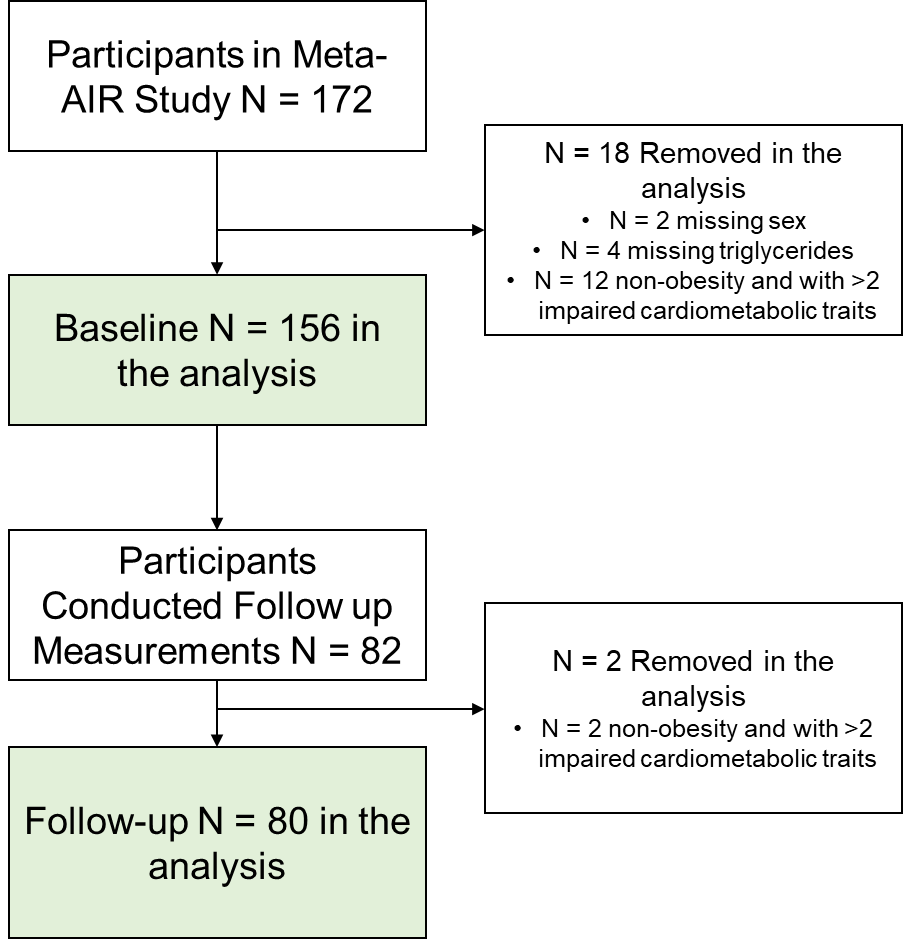

Supplement: Supplementary file 1 — Supplementary Figures. [file 41598_2024_57751_MOESM1_ESM.docx]
